# Acaricidal activity of ethanolic extract from aerial parts of *Tagetes patula* L. (Asteraceae) against larvae and engorged adult females of *Rhipicephalus sanguineus* (Latreille, 1806)

**DOI:** 10.1186/1756-3305-5-295

**Published:** 2012-12-17

**Authors:** Flávio Augusto Sanches Politi, Glyn Mara Figueira, Andréa Mendez Araújo, Bruno Rodrigues Sampieri, Maria Izabel Camargo Mathias, Matias Pablo Juan Szabó, Gervásio Henrique Bechara, Lourdes Campaner dos Santos, Wagner Vilegas, Rosemeire Cristina Linhari Rodrigues Pietro

**Affiliations:** 1 Department of Drugs and Medicines, School of Pharmaceutical Sciences, UNESP - Univ Estadual Paulista, Rodovia Araraquara-Jaú, Km 01, SP, Araraquara, CEP 14801-902, Brazil; 2 Chemical, Biological and Agricultural Pluridisciplinary Research Center (CPQBA), UNICAMP - Campinas State University, SP, Campinas, CP 6171, CEP 13081-970, Brazil; 3 Department of Biology, Institute of Biosciences, UNESP - Univ Estadual Paulista, Avenida 24-A, 1515, Bairro Bela Vista, SP, Rio Claro, CEP 13506-900, Brazil; 4 Department of Applied Immunology and Parasitology, Faculty of Veterinary Medicine, UFU - Federal University of Uberlândia, Avenida Pará 1720, MG, Uberlândia, CP 593, CEP 38400-902, Brazil; 5 Department of Veterinary Pathology, College of Agricultural and Veterinary Sciences, UNESP - Univ Estadual Paulista, Via de Acesso Professor Paulo Donato Castellane s/n, SP, Jaboticabal, CEP 14884-900, Brazil; 6 Department of Organic Chemistry, Chemistry Institute, UNESP - Univ Estadual Paulista, Rua Professor Francisco Degni 55, Bairro Quitandinha, SP, Araraquara, CEP 14800-900, Brazil

**Keywords:** *Tagetes patula*, *Rhipicephalus sanguineus*, flavonoids, mass spectrometry, adult immersion test, larval immersion test

## Abstract

**Background:**

The tick *Rhipicephalus sanguineus* is the species with the largest worldwide distribution and is proven to be involved in the transmission of pathogens such as *Babesia canis*, *Ehrlichia canis*, *Coxiella burnetii*, *Rickettsia ricketsii*, *Rickettsia conorii*, among others. Studies have demonstrated acquisition of resistance to some of the active principles used in commercial formulations of acaricides. *Tagetes patula* (Asteraceae) is a plant with highlighted economic and commercial importance due to the production of secondary metabolites with insecticide and acaricide potential, mainly flavonoids, thiophenes and terpenes.

**Methods:**

The *in vitro* acaricide action of the ethanolic 70% extract from aerial parts of *T. patula*, obtained by percolation, was evaluated against larvae and engorged adult females of *Rhipicephalus sanguineus* by immersion test for 5 minutes. The chemical characterization of this extract was done by liquid chromatography coupled with mass spectrometry (LC-MS), using direct injection of sample.

**Results:**

Despite *T. patula* not proving lethal to adults in any of the concentrations tested, at 50.0 mg/mL oviposition rate decreased by 21.5% and eliminated 99.78% of the larvae. Also it was determined that the best results were obtained with 5 minutes of immersion. From the chromatographic analysis twelve *O*-glycosylated flavonoids were identified.

**Conclusions:**

This is the first report on the acaricidal activity of *T. patula* extract against *Rh. sanguineus*. If we consider the application of the product in the environment, we could completely eliminate the larval stage of development of the ixodid *Rh. sanguineus.*

## Background

The intense agricultural activity, the interaction of man and domestic animals and the recent background of climate change in the world favor the spread of infectious agents transmitted by ticks, leading to the emergence and resurgence of different etiologic agents [1].

The subclass *Acari*, class *Arachnida*, which belongs to ticks and other mites, is a very heterogeneous group presenting great diversity of habits and habitats [2]. Ticks are inserted in the order *Ixodida*, which can be divided into three families: *Argasidae*, *Ixodidae* and *Nuttalliellidae*. The tick *Rhipicephalus sanguineus* (Latreille, 1806) is the species currently with the most worldwide spread, due the wide distribution of its natural host, the dog, and also due to nidicolous habits [3]. It is related to the transmission of pathogenic agents, mainly *Babesia canis* and *Ehrlichia canis*[4-6]. The parasite can also transmit several *Rickettsia* species to man, such as *Rickettsia rickettsii* and *R. conorii*, the causative agents, respectively, of Rocky Mountain spotted fever in Mexico and South America, and Botonous Fever in the Mediterranean region of Southern Europe and Northern Africa [7]. Fipronil, amitraz, carbaryl and pyrethroids (deltamethrin, permethrin and cypermethrin) are the acaricides most employed for its control [8-13], however, some authors have reported resistance of ticks to commercial formulations containing these compounds [14]. The use exclusive of synthetic products is becoming less viable in practical and economic terms, since its indiscriminate use causes environmental pollution, toxicity to humans and the appearance of chemical residues in products of animal origin [15]. When thinking about the control of *Rh. sanguineus* ticks, it must be considered that only 5% are found on dogs and the other 95% are free in the environment. Therefore, its effective elimination will require an integrated control strategy, aimed at both the canine population and the environment [16,17].

Plant extracts can be used to control certain species of ticks such as *Hyalomma anatolicum excavatum*[18], *Amblyomma americanum, Dermacentor variabilis*[19] and even *Rh. sanguineus*[20-23]. Among the advantages of phytotherapics that currently justify their use are the synergistic effects of its compounds, the combination of mechanisms for substances acting on different molecular targets, the lowest risk of side effects and lower costs in research [24].

*Tagetes patula* L. (Asteraceae), popularly known as dwarf marigold or French marigold, is an annual plant with 20–30 cm height, native to North America and widely disseminated worldwide [25]. Its flowers have varied coloration, and may present yellow petals, orange or a mix of these two shades. Is easily cultured and propagated, producing flowers and seeds throughout the year, with high germination rates. The phytochemical investigation of different parts of *T. patula* has resulted in the isolation of the chemical constituents of several classes of secondary metabolites, such as flavonoids, benzofurans, carotenoids and thiophenes, the latter being responsible for a variety of biocidal effects [26]. Bano *et al*. [27], from different parts of *T. patula* (roots, leaves and flowers) isolated and characterized by spectroscopic methods, several thiophenes, steroids and terpenoids: 5^′^- hydroxymethyl-5-(3-butene-1-ynil)-2,2^′^-bithiophene; methyl-5-[4-(3- methyl-1- oxobutoxy)-1-butynyl]-2,2’ bithiophene; cholesterol; β-sitosterol (24-R-stigmast-5-ene-3β-ol) (4); stigmasterol [24-(S)-stigmast-5,22E-dien-3β-ol] and lupeol. Flavonoids, such as kaempferol and quercetina, were reported by Ivancheva and Zdravkova [28]. Tarpo [29-31] and Bhardwaj *et al.*[32], recorded the presence of patuletin-7-*O*-glucoside (patulitrin), patuletin, quercetagetin, quercetagetin-7-*O*-glucoside and luteolin in petals of *T. patula*.

This study aimed to perform the chemical characterization by high pressure liquid chromatography coupled to mass spectrometry (HPLC-MS) of the 70% ethanolic extract from aerial parts of *T. patula* and test the acaricidal action in larvae and engorged adult females of *Rh. sanguineus* through immersion tests for 5 minutes.

## Methods

### Plant material

Aerial parts of *Tagetes patula* (stems, leaves and flowers) were obtained through an agreement signed with the Collection of Medicinal and Aromatic Plants (CPMA) of the Multidisciplinary Center for Chemical, Biological and Agricultural Research (CPQBA), Universidade Estadual de Campinas (UNICAMP). The planting was done in a total area of 100 m^2^, from seeds of Top Seed Garden line (Agristar®). The harvesting occurred in June 2010. A voucher specimen was deposited under number 1421 in the CPQBA Herbarium.

### Sample preparation

After the stabilization and drying, the aerial parts of the plant were triturated into cutting mill. The powdered drug was used for preparing the extract by percolation using ethanol 70% (v/v) as the extractor liquid, with average flow rate of 40 drops/minute. After complete evaporation of the solvent, this extract was lyophilized and stored in a desiccator to avoid incorporating humidity and/or contamination.

For the chemical characterization by liquid chromatography coupled to mass spectrometry, the extract was solubilized in MeOH (Baker®, HPLC grade) to give a solution 1.0 mg/ml, which was filtered in expanded polytetrafluoroethylene membrane (PTFE) with pores of 0.45 μm. The final solution was introduced directly into the ESI source using a glass syringe boosted by a pumping system with outflow of 20.0 mL/min.

The solution used in the *Adult Immersion Test* (AIT) and *Larval Immersion Test* (LIT) was prepared using Triton-X-100 1.25% (v/v), because ethanol proved toxic in preliminary tests. Serial dilution was prepared so as to obtain test solutions 12.5, 25.0, 50.0 and 100.0 mg/mL.

### Collection of ticks

Engorged adult females of *Rh. sanguineus* were obtained from the colony of the *Brazilian Centre of Studies on Tick Morphology* (BCSTM), Universidade Estadual Paulista "Julio de Mesquita Filho" (UNESP), Instituto de Biociências (IB), Rio Claro (SP), maintained under controlled conditions (27–28°C, 70-80% RH, 12/12 h photoperiod) in BOD incubator (Eletrolab EL 202/3). These ticks were fed in fabric chambers on the dorsum of New Zealand white rabbits (*Oryctolagus cuniculis*) [approved by the ethics committee on the use of animal (CEUA), IB-UNESP, protocol number 026/2011], according to the procedure described by Bechara *et al.*[33].

### Mass spectrometry

The mass spectra of the ethanolic 70% extract of aerial parts of *T. patula* were obtained on a HPLC coupled to a mass spectrometer LCQ Fleet (Thermo Scientific®), equipped with a dispositive of direct insertion of the sample via flow injection analysis (FIA). The studied matrix was analyzed by electrospray ionization (ESI) and the fragmentation in multiple stages (MS^2^, MS^3^, MS^n^) was performed at an ion trap (IT) interface. The negative mode was selected for the generation and analysis of the mass spectra for the first order (MS), and for the remaining experiments in multiple stages (MS^n^) under the following conditions: capillary voltage −25 V, voltage spray −5 kV, capillary temperature 275°C, carrier gas nitrogen (N_2_) with a flow of 8 arbitrary units (A.U.), collision gas helium (He). The track acquisition was 100–2000 *m/z*. The software Xcalibur version 1.3 (Thermo Finigan®) was used to acquire and process data.

### Adult Immersion Test (AIT)

Groups of 10 *Rhipicephalus sanguineus* engorged females, chosen randomly, were immersed for 5 minutes in Petri dishes (5.5 cm diameter, 1.5 cm high) containing 10.0 mL of the respective dilutions of 70% ethanolic extract from aerial parts of *T. patula* (AP_EtOH70%_): 12.5, 25.0, 50.0 and 100.0 mg/mL, using a solution of Triton X-100 1.25% (v/v) as negative control. The ticks were removed from solution, dried on paper towels and gently allocated individually in sterile 24-well plates, incubated at 27–28°C and 70-80% RH (12/12 h photoperiod) in a BOD incubator. After two weeks it was determined the number of females laying eggs. The eggs were collected, weighed and then placed in glass tubes, which were incubated under the same conditions described above [34].

The calculation of the percentage of egg laying inhibition was performed according to equations [35]:

(1) $$\begin{array}{l} \mathrm{IE}\left(\mathrm{index}\;\mathrm{of}\;\mathrm{egg}\;\mathrm{laying}\right)\\ \;=\frac{\mathrm{weight}\;\mathrm{of}\;\mathrm{eggs}\;\mathrm{laid}\;\left(g\right)}{\mathrm{weight}\;\mathrm{of}\;\mathrm{females}\;\left(g\right)} \end{array}$$

(2) $$\begin{array}{l} \mathrm{Egg}\;\mathrm{laying}\;i\mathrm{nhibition}\;\left(\%\right)\\ \;=\frac{\left[\mathrm{IE}\left(\mathrm{control}\right)-\mathrm{IE}\;\left(\mathrm{treated}\;\mathrm{group}\right)\right]\times 100}{\mathrm{IE}\left(\mathrm{control}\right)} \end{array}$$

At the end of the third week the larval hatching rate was estimated, performing the count using a stereoscope. The efficiency of the extract was calculated according to the equations proposed by Drummond *et al.*[36]:

(3) $$\mathrm{RE}=\frac{\left[\mathrm{weight}\;\mathrm{of}\;\mathrm{eggs}\left(g\right)\times \mathrm{percentage}\;\mathrm{of}\;\mathrm{hatching}\times 20,000*\right]}{\mathrm{weight}\;\mathrm{of}\;\mathrm{female}\left(g\right)}$$

(4) $$\mathrm{PE}=\frac{\left[\mathrm{RE}\left(\mathrm{control}\;\mathrm{group}\right)-\mathrm{RE}\left(\mathrm{treated}\;\mathrm{group}\right)\times 100\right]}{\mathrm{RE}\left(\mathrm{control}\;\mathrm{group}\right)}$$

Where: ER = reproductive efficiency; = EP product efficiency; * = number of larvae at approximately 1.0 g of eggs (number experimentally obtained).

### Kinetics of action of the extract AP_EtOH70%_ in the adult immersion test

In order to determine the correspondence of the exposure time of ticks in the test solution with the results obtained, the *Adult Immersion Test* with engorged females in 70% ethanolic extract from aerial parts of *T. patula* (50.0 mg/ml) for 5, 10, 30 and 60 minutes was used. The test conditions were identical to those described above for *t* = 5 minutes. Results represent the average of two assays, with groups containing five ticks each.

### Larval Immersion Test (LIT)

**Figure 1:**
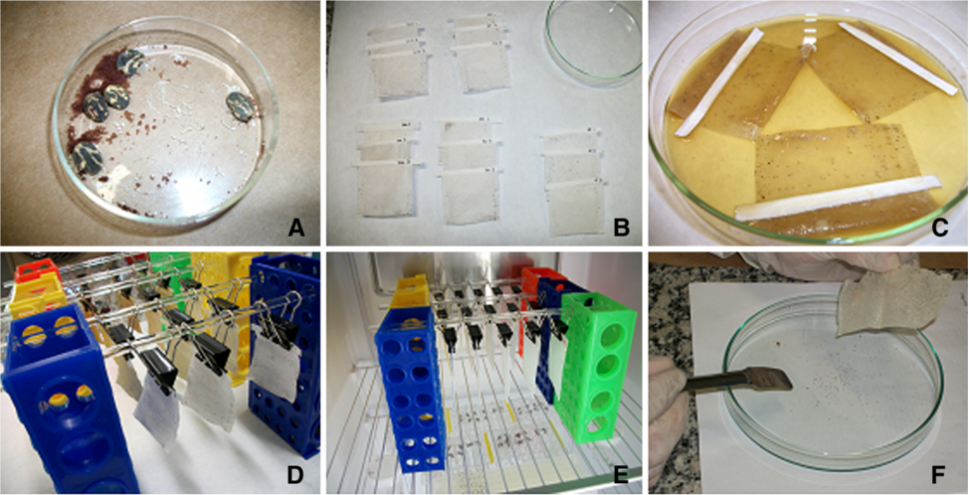
**Experimental procedure for the Larval Immersion Test.** (**A**-**B**) Approximately 1.0 g of eggs were collected and transferred individually to bags of TNT fabric; (**C**) these bags were immersed for 5 minutes in dilutions of 70% ethanolic extract of *T. patula*; (**D**-**E**) the bags were dried at room temperature and taken to BOD (27–28°C, 70-80% RH, photoperiod 12/12 h), (**F**) After 3 weeks, living and dead larvae were counted.

The test was performed in triplicate following the protocol proposed by Shaw [37] with modifications. Approximately 0.01 g of eggs were placed individually in little bags of TNT fabric (6.0 cm x 6.0 cm) and incubated in a BOD incubator at 27–28°C and 70-80% RH (12/12 h photoperiod) for three weeks. After this period of hatching, approximately 200 viable larvae were collected and transferred to new bags. The procedure was to place the contents of the package, previously cooled to −8°C for 1 minute, in the center of a Petri dish, kept above a vessel containing water and detergent, to prevent the escape of larvae in the laboratory. Under the effect of low temperature to which they were subjected, the larvae remained temporarily paralyzed, allowing their relocation. These new bags containing the larvae were immersed for 5 minutes in Petri dishes with 20.0 ml of the respective dilutions of the extract AP_EtOH70%_: 12.5, 25.0, 50.0 and 100.0 mg/mL, using distilled water as negative control. Then, the bags were left on the filter paper until completely dry. Following this procedure, the bags were incubated at 27–28°C and 70-80% RH (12/12 h photoperiod) for 48 hours (Figure 1). Live and dead larvae were counted to calculate the mortality rate, corrected according to Abbott's formula [38], recommended by the Food Agriculture Organization of the United Nations (FAO) [39]:

(5) $$\%\mathrm{Mortality}\left(\mathrm{corrected}\right)=\frac{\left[\%\;\mathrm{Mortality}\left(\mathrm{test}\;\mathrm{group}\right)-\%\mathrm{Mortality}\left(\mathrm{control}\;\mathrm{group}\right)\right]}{\left[100\;-\;(\%)\;\mathrm{Mortality}\left(\mathrm{control}\;\mathrm{group}\right)\right]\times 100}$$

### Statistical analysis

All statistical analyses were performed with StatPlus 2009 software (Soft Analyst®). Results in *Adult Immersion Test* (mortality, egg laying inhibition, percentage of hatching, product efficiency, among other parameters) were analyzed by Sheffé, Tukey-Kramer and Neuman-Keuls tests (significance level: p < 0.05). Lethal concentrations (LC) to kill 50% and 99% of larvae and their respective 95% confidence intervals (CI) were calculated by probit analysis.

## Results

**Figure 2:**
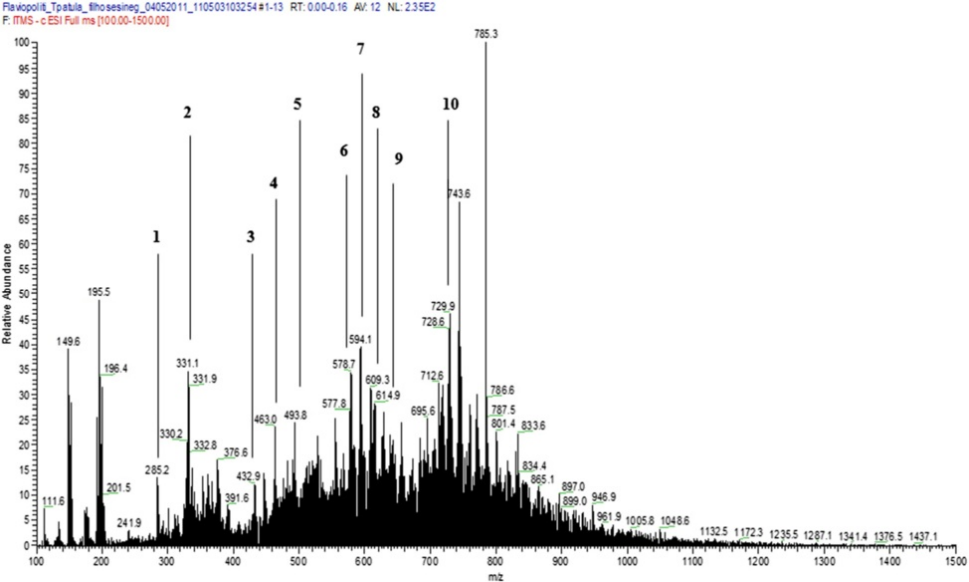
**Chromatogram analysis by LC/FIA-ESI-IT-MS of 70% ethanolic extract of the aerial parts from *T. patula.*** To spectrometric conditions detailed see the text.

The second order fragmentation (MS/MS) for each of the most representative ions led to the identification of secondary metabolites, observing the presence of flavonoids *O*-glycosides (Table 1), very common in the genus *Tagetes*[40-44]. Figure 2 shows the mass spectrum of the 70% ethanolic extract of the aerial parts of *T. patula* (AP_EtOH70%_) obtained by direct injection in negative ionization mode (*m/z* [MH]^-^).

**Table 1:** **List of the phenolic compounds identified in*Tagetes patula*by LC/ESI-IT-MS**

**Compound**	**Peak**	**[M-H]**^**-**^	**Product ion scan fragments of [M-H]**^**-**^
Kaempferol	1	285	151, 134
Patuletin	2	331	316
Quercetin-3-*O*-pentoside	3	433	316
Quercetin-3-*O*-glucoside (isoquercitrin) or Quercetina-3-*O*-galactoside (hyperoside)	4	463	301
Patuletin-7-*O*-glucoside (patulitrin) or 6-*O*-methyl-quercetin-3-*O*-glucoside	5	493	331, 316
Quercetin-3-*O*-rhamnosyl-*O*-xyloside	6	579	447, 301
Quercetin-3-*O*-di-rhamnoside	7	593	447, 301
Quercetin-3-*O*-glycosyl-7-*O*-rhamnosyl	8a	609	447, 301
Quercetin-3-*O*-rhamnosyl-7-*O*-glycosyl	8b	609	463, 301
Kaempferol-3-*O*-di-hexoside	8c	609	447, 285
Quercetin-3-*O*-hexoside-galloyl	9	615	463, 301
Kaempferol-3-*O*-rhamnoside-galloyl	10	729	583, 431

Peak 1 showed the signal of a deprotonated molecule [MH]^-^ in *m/z* 285. The fragmentation of second order was not stable, however, from the analysis of the fragments presented in full scan spectra (MS^2^), the retro-Diels-Alder reaction (RDA) could be observed, with pattern of fragmentation of ions at *m/z* 151 and at *m/z* 134, suggesting that this substance is kaempferol [45].

Peak 2 showed the signal of a deprotonated molecule [MH]^-^ at *m/z* 331. The second order fragmentation of precursor ion of *m/z* 331 generated the ion product *m/z* 316 [M-CH_3_-H]^-^. These data, together with literature references [43], allowed identification of the compound as patuletin.

Peak 3 exhibited the signal of a deprotonated molecule [MH]^-^ at *m/z* 433. The second order fragmentation generated the ion product *m/z* 316 [M-H-132]^-^, corresponding to loss of one pentose unit, suggesting it was quercetin-3-*O*-pentoside, compatible with quercetin-3-*O*-xylose isolated in *T. maxima*[43].

Peak 4 presented the signal of a deprotonated molecule [MH]^-^ at *m/z* 463. The second order fragmentation generated the ion product *m/z* 301 [M-H-162]^-^, corresponding to loss of one hexose unit, suggesting it was quercetin-3-*O*-glucoside (isoquercitrin) or quercetin-3-*O*-galactoside (hyperoside).

Peak 5 exhibited the signal of a deprotonated molecule [MH]^-^ at *m/z* 493. The second order fragmentation generated the signal *m/z* 331 [M-H-162]^-^ and the signal of *m/z* 316 [M-CH3-H]^-^, suggesting, according to data from the literature [43], that the compound could be patuletin-7-*O*-glucoside (patulitrin) or 6-*O*-methyl-quercetin-3-*O*-glucoside.

Peak 6 signal showed the signal of a deprotonated molecule [MH]^-^ at *m/z* 579. The second order fragmentation with precursor ion at *m/z* 579 generated the signal of *m/z* 447 [M-H-132]^-^, attributed to the loss of a xylose unit. Fragmentation of the ion at *m/z* 447 generated the signal *m/z* 301 [M-H-146]^-^, corresponding to loss of one deoxyhexose unit (*e.g.* rhamnnose). Considering the sequence the loss of sugars led to the proposal that the structure was of quercetin-3-*O*-rhamnosyl-*O*-xyloside for this substance.

Peak 7 exhibited the signal of a deprotonated molecule [MH]^-^ at *m/z* 593. The second order fragmentation generated the signals *m/z* 447 [M-H-146]^-^ and *m/z* 301 [M-H-146]^-^, corresponding to the loss of two deoxyhexose units in sequence, suggesting it was quercetin-3-*O*-di-rhamnoside.

Peak 8 showed the signal of a deprotonated molecule [MH]^-^ at *m/z* 609. The second order fragmentation generated three possibilities: signals *m/z* 447 [M-H-162]^-^ and *m/z* 301 [M-H-146]^-^, corresponding to successive loss of a hexose unit and a deoxyhexose unit, suggesting it was quercetin-3-*O*-glycosyl-7-*O*-ramnosyl; signals *m/z* 463 [M-H-146]^-^ and *m/z* 301 [M-H-162]^-^, corresponding to successive loss of a deoxyhexose unit and a hexose unit, suggesting it could be quercetin-3-*O*-ramnosyl-7-*O*-glucosyl and signals at *m/z* 447 [M-H-162]^-^ and *m/z* 285 [M-142-H]^-^, corresponding to successive loss of two hexose units, suggesting it could be kaempferol-3-*O*-di-hexoside.

Peak 9 exhibited the signal of a deprotonated molecule [MH]^-^ at *m/z* 615. The second order fragmentation generated signals *m/z* 463 [M-H-152]^-^ and *m/z* 301 [M-H-162]^-^, corresponding to sequential loss of one galloyl unit and one hexose unit, suggesting it could be quercetin-3-*O*-hexoside-galloyl.

Peak 10 showed the signal of a deprotonated molecule [MH]^-^ at *m/z* 729. The second order fragmentation generated signals at *m/z* 583 [M-H-146]^-^ and *m/z* 431 [M-H-152]^-^, relating to sequential losses of one rhamnose unit and one galloyl unit. These data suggest that the compound may be kaempferol-3-*O*-rhamnoside-galloyl.

Mortality of engorged adult females of *Rh. sanguineus* subjected to 70% ethanolic extract was very low. There was only 1 death in the 12.5 mg/mL group (5%), 1 death in the 25.0 mg/mL group (5%), 2 deaths in the 50.0 mg/mL group (10%) and 1 death in the 100.0 mg/mL group (5%). With this reduced number of deaths, the LC_50_ was not estimated (lethal concentration to 50% of organisms), because a very high value would be obtained, probably toxic to dogs and unattractive from a commercial standpoint. Table 2 shows the values of the percentage inhibition of egg laying, referring to the average of two assays.

**Table 2:** **Index of egg laying and percentage of egg laying inhibition in females of*Rhipicephalus sanguineus***

**Samples**	**Weight of the Ticks (g)**	**Weight of the Eggs (g)**	**IE**^**1**^	**Egg Laying Inhibition (%)**
AP_EtOH70%_	0.1947^a^	0.1133^a^	0.5815^a^	7.0794^b^
(12.5 mg/mL)	(± 0.010)	(± 0.010)	(± 0.024)	(± 3.414)
AP_EtOH70%_	0.1841^a^	0.1032^a^	0.5625^a^	11.4363^b^
(25.0 mg/mL)	(± 0.028)	(± 0.011)	(± 0.025)	(± 1.114)
AP_EtOH70%_	0.1858^a^	0.0932^a^	0.5024^a^	21,5088^c^
(50.0 mg/mL)	(± 0.020)	(± 0.008)	(± 0.009)	(± 2.151)
AP_EtOH70%_	0.1822^a^	0.1011^a^	0.5577^a^	12.0528^b^
(100.0 mg/mL)	(± 0.045)	(± 0.020)	(± 0.023)	(± 1.665)
Triton-X-100	0.1768^a^	0.1120^a^	0.6338^a^	0.0^a^
(1.25%, v/v)	(± 0.004)	(± 0.004)	(± 0.039)	(± 0.0)

^1^ IE: index of egg laying; values with the same superscript letters within a column do not show statistically significant differences by Sheffé, Tukey-Kramer and Neuman-Keuls tests (p < 0.05). The results represent the mean of two assays.

Table 2 reveals that there was an increased percentage inhibition of egg laying at the lowest concentrations (12.5 and 25.0 mg/ml) until 50.0 mg/mL, from which the increase of concentration resulted in decreased activity to about half, showing a mathematical pattern expressed by the following equation: y = − 0.006x^2^ + 0.787x - 2.663 (R^2^ = 0.964), where *y* = percentage of inhibition of egg laying and *x* = concentration of test sample. There were no statistically significant differences between the weight of the animals and their egg weights, avoiding bias in the results.

The percentage hatching of larvae was calculated assuming that 1.0 g of eggs correspond to approximately 20,000 larvae, mean value obtained from egg masses (without any chemical treatments) presenting hatching rate above 95%. Considering this, and based on the weight of eggs laid per treatment, the expected number of hatched larvae was obtained. Counting the total number of larvae per glass tube, provided the actual number of hatched larva and allowed an estimation of the percentage of larvae hatching. Table 3 presents the results for the average of two assays (± standard deviations) of reproductive efficiency calculating (RE) and product efficiency (PE) to dilutions of the 70% ethanolic extract of the aerial parts of *T. patula* (AP_EtOH70%_). The greater effectiveness of the product was obtained to the extract AP_EtOH70%_ at a concentration of 50.0 mg/mL (PE = 42.45%) and the lowest effectiveness at concentration of 12.5 mg/mL (PE = 25.29%). Regarding the percentage of hatching for all concentrations tested showed values below the control (p < 0.05).

**Table 3:** **Values of reproductive efficiency and product efficiency of 70% ethanolic extract from*Tagetes patula***

**Samples**	**W**_**eggs**_^**1**^**(g)**	**H**_**eggs**_^**2**^**(%)**	**RE**^**3**^	**PE**^**4**^**(%)**
Triton-X-100	0.1120^a^	92.0^a^	210569.4^a^	**-**
(1.25%, v/v)	(± 0.004)	(± 2.828)	(± 7989.45)	
AP_EtOH70%_	0.1133^a^	71.42^b,c^	157525.71^b^	25.29^a^
(12.5 mg/mL)	(± 0.010)	(± 3.818)	(± 17520.79)	(± 5.486)
AP_EtOH70%_	0.1094^a^	57.04^b^	123639.12^b^	41.14^a^
(25.0 mg/mL)	(± 0.020)	(± 5.798)	(± 10222.45)	(± 7.087)
AP_EtOH70%_	0.9875^a^	61.89^b,c^	121167.47^b^	42.45^a^
(50.0 mg/mL)	(± 0.016)	(± 6.639)	(± 6931.52)	(± 3.291)
AP_EtOH70%_	0.1011^a^	76.83^c^	154654.66^b^	26.55^a^
(100.0 mg/mL)	(± 0.020)	(± 3.323)	(± 25441.67)	(± 12.082)

^1^ W_eggs_: total weight of eggs laid; ^2^ H_eggs_: percentage of hatching of eggs laid; ^3^ RE: reproductive efficiency, ^4^ PE: efficiency of the product (percentage). Values with the same superscript letters within a column do not showed statistically significant differences by Tukey and Neuman-Keuls tests (p < 0.05).

**Figure 3:**
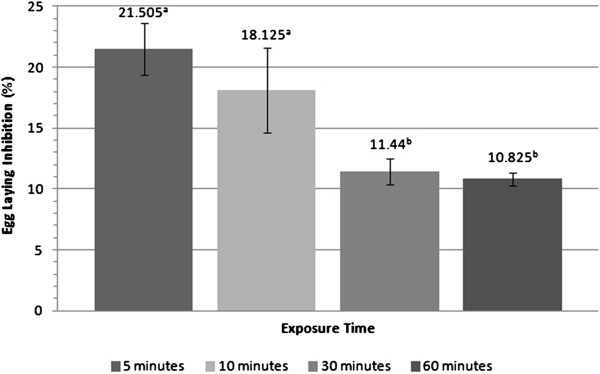
**Action of 70% ethanolic extract from aerial parts of *T. patula *(50.0 mg/mL) in engorged female adult of *Rh. sanguineus *in the *Adult Immersion Test *for 5, 10, 30 and 60 minutes.** Values with the same superscript letters do not show statistically significant differences by Tukey and Neuman-Keuls tests (p < 0.05).

Figure 3 shows the results of the kinetic test of activity to the extract AP_EtOH70%_. The values obtained at 5 and 10 minutes were statistically similar, but differ from the values at 30 and 60 minutes, which were similar to each other. The results demonstrate that a longer immersion time of ticks in the extract solution does not potentiate its action, on the contrary, it decreases the percentage of egg laying. There were no reported cases of deaths of adult females after contact with 70% ethanolic extract from aerial parts of *T. patula*, in any groups tested.

Table 4 shows the results of Larval Immersion Test. It can be observed that at concentrations of 50.0 mg/mL and 100.0 mg/mL, there was approximately 100% killing of the larvae sampled. Even at the lowest concentration (12.5 mg/mL), the extract showed efficiency above 70%.

**Table 4:** **Larval mortality rate of*Rhipicephalus sanguineus*front dilutions of 70% ethanolic extract of*Tagetes patula***

**Samples**	**Number of living larvae**	**Number of death larvae**	**(%) Mortality**^**1**^
Distilled Water	145.33^a^	17.00^a^	10.48^a^
	(± 24.110)	(± 2.645)	(± 0.342)
AP_EtOH70%_	35.66^b^	107.66^b^	72.26^b^
(12.5 mg/mL)	(± 6.429)	(± 16.072)	(± 0.628)
AP_EtOH70%_	46.33^b^	134.00^b^	71.35^b^
(25.0 mg/mL)	(± 8.020)	(± 19.697)	(± 1.344)
AP_EtOH70%_	0.33^c^	178.33^c^	99.78^c^
(50.0 mg/mL)	(± 0.577)	(± 17.897)	(± 0.367)
AP_EtOH70%_	0.33^c^	108.666^b^	99.72^c^
(100.0 mg/mL)	(± 0.577)	(± 27.006)	(± 0.472)

^1^ The mortality rate was calculated using Abbott's formula, due to the mortality rate in the control group was close to 10.0%. Values with the same superscript letters within a column do not show statistically significant differences by Sheffé, Tukey-Kramer and Neuman-Keuls tests (p < 0.05). The values are the mean of three assays, ± the standard deviation.

The larval IC_50_ was calculated from the probit analysis (Finney method) with the mortality rate data, obtaining the value of 7.43 mg/mL, with 95% level of significance (Table 5).

**Table 5:** **Lethal concentrations of 70% ethanolic extract of*Tagetes patula*in larvae of*Rhipicephalus sanguineus***

**LC**^**1**^**(Percentile)**	**LC**_**AP**_^**2**^**(mg/mL)**	**Level of 95% of significance**
LC_01_	0.51	(0.29 – 0.77)
LC_10_	1.70	(1.57 – 2.24)
LC_25_	3.42	(3.34 – 4.12)
LC_50_	7.43	(7.42 – 7.68)
LC_90_	32.51	(32.45 – 37.39)
LC_95_	49.40	(49.35 – 55.19)
LC_99_	108.25	(108.19 – 123.67)

Chi-square (*X*^2^) = 4.2096; p < 0.05; p-level = 0.1219; ^1^ LC: lethal concentration percentile; ^2^ LC_AP_: lethal concentration values of 70% ethanolic extract of aerial parts of *T. patula* calculated from the interpolation of the mortality results, by probit analysis (Finney method).

## Discussion

The use of natural products in the control of brown dog tick *Rh. sanguineus* has been the focus of research in many countries, especially with the increased resistance that these organisms present to commercial acaricides [14]. The main mechanisms used by resistant ticks to survive against acaricides are: reduction of the penetration rate of the product by changing the outer tegument; changes in metabolism, storage and excretion of the acaricide and changes of the site of action of chemical products [46]. In Brazil, studies that use plant extracts or essential oils against these ticks have been increasingly recurrent, some of them showing promising results.

The coupling of liquid chromatography and mass spectrometry is often used to characterize the chemical constituents of plant extracts. The mass spectrometer is considered as a universal detector, and thus the union brings a time saving element in the analysis of a complex matrix, because it avoids the isolation of its chemical constituents. Besides the chromatographic separation and the information on the molecular weight of substances, fragments of the molecules in question are also obtained, which are important for the structural elucidation of different classes of secondary metabolites [47]. Due to the high sensitivity, this system can detect minor compounds that have been isolated by standard phytochemical techniques [48]. The second order fragmentation (MS/MS) for each of the most representative ions led to the identification of *O*-glycosylated flavonoids. In this type of flavonoid the *O-C* bond is more susceptible to cleavage. The break and concomitant rearrangement of the hydroxyl hydrogen, leads to removal of monosaccharides, with losses of 176 mass units (uronic acid), 162 mass units (hexose), 146 mass units (deoxyhexose) or 132 mass units (pentose), allowing determination of the sequence of carbohydrates present in the aglycones [49]. In this work, when compared to positives, the negative mode led to results more elucidative to obtain the chemical composition of the extract of *T. patula*. Thus, although the positive mode has been evaluated, only the results in negative mode were presented, giving a total of 12 possible compounds in the constitution of the 70% ethanolic extract of the aerial parts from *T. patula*. These substances are in agreement with records from the literature, which describe the presence of phenolic compounds in various species of *Tagetes*[41-43,50-52].

In this study, the immersion test of engorged adult *Rh. sanguineus* showed no significant rates of death, occurring only in a few isolated cases in the replicates of each test. The most effective concentration tested was 50.0 mg/mL, showing a percentage of inhibition of egg laying of 21.50%. No previous reports testing natural plant extractives against engorged adult females of *Rh. sanguineus* were found in the literature, precluding comparison with the results presented in this study, making it the pioneer. In a study with *Riphicephalus (Boophilus) microplus*, Ribeiro *et al.*[53] found as best result of percentage of egg laying inhibition the value of 19.2% for hexane extract of *Hypericum polyanthemum* (Guttiferae) (25.0 mg/mL). In another investigation, Ribeiro *et al*. [34] found values of percentage of egg laying inhibition of *R. (Bophilus) microplus* ranging between 11.7% and 14.6%, using hexane extract of *Calea serrata* (Asteraceae).

The efficacy of the extracts was determined according to estimated reproduction calculation proposed by Drummond *et al*. [36]. The reproductive rate is influenced by several factors, the main ones being the temperature and relative humidity. In *in vitro* experiments these factors are controlled, which means that the results obtained are only due to the treatments. The analysis of variance (ANOVA) shows that the values of PE showed no statistically significant differences between groups. Furthermore, the results presented in Table 2 revealed a pattern of action of samples. It was found that there is similarity in the percentage of egg hatchability and in the product efficiency between extreme dilutions (12.5 mg/mL and 100.0 mg/mL) and between the intermediate dilutions (25.0/mL and 50.0 mg/mL). Thus, it can be suggested that the efficiency in the inhibition of posture and/or hatching of the eggs is dose-dependent, i.e., from an optimal concentration, the inhibition rates tend to decay relatively. Overall, the 70% ethanolic extract of the aerial parts of *T. patula* (AP_EtOH70%_) to 50.0 mg/mL was the most efficient, with a value of PE = 42.45%. Broglio-Micheletti *et al.*[54] obtained values of product efficiency ranging between 18.35% to 2% ethanolic fraction of leaves of *Cymbopogon citratus* (Poaceae) and 59.24% to 2% ethanolic fraction of flowers of *Syzygium malaccensis* (Myrtaceae).

When the exposure time of the ticks in the solutions tested was varied, statistically significant differences between the minimum and maximum were found. Interestingly, the percentage of egg laying inhibition of the group immersed for 5 minutes was almost twice that found in the group immersed for 60 minutes (21.5% and 10.82%, respectively). Further study of the mechanism of action of the extract AP_EtOH70%_ should be conducted to explain this finding, however, it can be suggested that 5 minutes is sufficient to obtain the results reported here and increments in the immersion time does not enhance its effectiveness.

Larval immersion tests showed very promising results. The 70% ethanolic extract of aerial parts of *T. patula* at 50.0 mg/mL and 100.0 mg/mL had mortality rates close 100%. Even at the lowest concentration (12.5 mg/mL), the extract showed mortality rates higher than 70%. Ribeiro *et al*. [53] obtained similar results, however, against *Rh. (Boophilus) microplus*. Using methanolic extract of *Hypericum polyanthemum* in concentrations of 50.0, 25.0, 12.5 and 6.25 mg/ml were obtained mortality rates of 100%, 96.7% (± 1, 5), 84.7% (± 3.5) and 52.7% (± 2.5), respectively.

In this study, 70% ethanolic extract of the aerial parts of *T. patula* presented larval LC_50_ and larval LC_95_ of 7.43 mg/mL and 49.4 mg/mL, respectively. Fernandes *et al.*[20], performing another type of assay, the classical larval packet test [39], found for ethanolic extract from stem bark of *Magonia pubescens* (Sapindaceae) LC_50_ and LC_99_ against larvae of *Rh. sanguineus* of 1.50 mg/mL and 9.99 mg/mL, respectively.

Briefly, in our study it was observed that low mortality rates caused by the tested extract on engorged adult forms and a lethal action close to 100% against larval forms. This discrepancy could be explained by the difference in the composition of the cuticle of both life stages of ticks. According Balashov [55], the cuticle of the ticks is formed by the outer layer, epicuticle (composed by waxes externally and by proteins internally) and the inner layer, called procuticle (protein and chitin). According Odhiambo [56], the layer of waxes or lipids is seen only from ecdysis in nymph and in greater quantity in the adult. Therefore, in engorged females, the solvents must first dissolve the lipid layer of the epicuticle in order to achieve the most polar layers of the cuticle, which consist of soluble proteins.

## Conclusions

The 70% ethanolic extract of aerial parts of *T. patula* 50 mg/ml reduced egg laying in 21.5% and eliminated 99.78% of the larvae (LC_50_ = 7.43 mg/mL), although it was not effective on mortality of engorged female adult of *Rh. sanguineus*. Also it was determined that the best results were obtained with 5 minutes of immersion. Further studies should be conducted in order to verify the *in vitro* cytotoxicity of this extract, however, the results reported here are important because it is a pioneering investigation and has suggested a novel methodology using the larval packet test. If we consider the application of the product in the environment, we would completely eliminate one of the developmental stages of the ixodid *Rh. sanguineus.*

## Competing interests

The authors declare that they have no competing interests.

## Authors’ contributions

FASP carried out the studies, the statistical analysis and drafted the manuscript. GMF provided the plant samples. AMA and BRS helped in maintaining the colony of ticks and preparation of experiments. MPJS, GHB and MICM participated in the design of the study with *Rh. sanguineus* and discussion of results. LCS and WV participated in the planning of chromatographic tests and discussion of data. RCLRP conceived of the study, and participated in its design and coordination, discussion of data, helped to draft the manuscript. All authors read and approved the final manuscript.
